# Urate-lowering therapy following a treat-to-target continuation strategy compared to a treat-to-avoid-symptoms discontinuation strategy in gout patients in remission (GO TEST Finale): study protocol of a multicentre pragmatic randomized superiority trial

**DOI:** 10.1186/s13063-023-07242-y

**Published:** 2023-04-19

**Authors:** Iris Rose Peeters, Alfons A. den Broeder, William J Taylor, Nathan den Broeder, Marcel Flendrie, Noortje van Herwaarden

**Affiliations:** 1 Department of Rheumatology, Sint Maartenskliniek, Ubbergen, the Netherlands; 2 Radboud Institute of Health Sciences, Radboud University Medical Center, Nijmegen, the Netherlands; 3 Department of Rheumatology, Radboud University Medical Center, Nijmegen, the Netherlands; 4 Department of Medicine, University of Otago Wellington, Newtown, Wellington, New Zealand; 5 Department of Pharmacology, Radboud University Medical Center, Nijmegen, the Netherlands

**Keywords:** Gout, Urate-lowering therapy, Remission, Treat-to-target strategy, Treat-to-avoid-symptoms strategy, Randomized clinical trial

## Abstract

**Background:**

Long-term gout treatment is based on reducing serum urate levels using urate-lowering therapy (ULT). Most guidelines recommend using a lifelong continuation treat-to-target (T2T) strategy, in which ULT is dosed or combined until a serum urate target has been reached and maintained. However, a frequently used alternative strategy in clinical practice is a treat-to-avoid-symptoms (T2S) ULT discontinuation strategy, with the possibility of restarting the medication. This latter strategy aims at an acceptable symptom state, regardless of serum urate levels. High-quality evidence to support either strategy for patients in prolonged remission while using ULT is lacking.

**Methods:**

We developed an investigator-driven pragmatic, open-label, multicentre, randomized, superiority treatment strategy trial (GO TEST Finale). At least 278 gout patients using ULT who are in remission (>12 months, preliminary gout remission criteria) will be randomized 1:1 to a continued T2T strategy (treatment target serum urate < 0.36 mmol/l) or switched to a T2S discontinuation strategy in which ULT is tapered to stop and restarted in case of (persistent or recurrent) flaring. The primary outcome is the between-group difference in the proportion of patients not in remission during the last 6 months of 24 months follow-up and will be analyzed using a two proportion *z* test. Secondary outcomes are group differences in gout flare incidence, reintroduction or adaptation of ULT, use of anti-inflammatory drugs, serum urate changes, occurrence of adverse events (with a special interest in cardiovascular and renal events), and cost-effectiveness.

**Discussion:**

This study will be the first clinical trial comparing two ULT treatment strategies in patients with gout in remission. It will contribute to more specific and unambiguous guideline recommendations and improved cost-effectiveness of long-term gout treatment. It also paves the way (exploratory) to individualized long-term ULT treatment. In this article, we elaborate on some of our trial design choices and their clinical and methodological consequences.

**Trial registration:**

International Clinical Trial Registry Platform (ICTRP) NL9245. Registered on 2 February 2021 (METC Oost-Nederland NL74350.091.20); EudraCT EUCTR2020-005730-15-NL. Registered on 11 January 2021.

## Administrative information

Note: the numbers in curly brackets in this protocol refer to SPIRIT checklist item numbers. The order of the items has been modified to group similar items (see http://www.equator-network.org/reporting-guidelines/spirit-2013-statement-defining-standard-protocol-items-for-clinical-trials/).

**Table:** 

Title {1}	Urate-lowering therapy following a treat-to-target continuation strategy compared to a treat-to-avoid-symptoms discontinuation strategy in gout patients in remission (GO TEST Finale): study protocol of a multicentre pragmatic randomized superiority trial.
Trial registration {2a and 2b}.	International Clinical Trial Registry Platform (ICTRP): NL9245, date of registration 2 February 2021 (METC Oost-Nederland NL74350.091.20), EudraCT: EUCTR2020-005730-15-NL, date of registration 11 January 2021.
Protocol version {3}	23-01-2023, version 11 (amendments of adding participating centers).
Funding {4}	The GO TEST Finale is funded by ZonMw (grant number: 80-87600-98-19030). During the grant proposal process the study protocol has been peer reviewed by external reviewers. After awarding the grant, ZonMw had not influenced the study design and/or data collection.
Author details {5a}	Peeters Iris Rose^1,2*^, den Broeder Alfons A ^1,4^, Taylor William J ^3^, den Broeder Nathan^1,2^, Flendrie Marcel^1^, van Herwaarden Noortje^1,5^ 1. Department of Rheumatology, Sint Maartenskliniek, Ubbergen, the Netherlands 2. Radboud Institute of Health Sciences, Radboud university medical centre, Nijmegen, the Netherlands 3. Department of Medicine, University of Otago Wellington, Newtown, Wellington, New Zealand 4. Department of Rheumatology, Radboud university medical center, Nijmegen, the Netherlands. 5. Department of Pharmacology, Radboud university medical center, Nijmegen, the Netherlands ^*^ Corresponding author: i.peeters@maartenskliniek.nl IRP, NdB, WT, MF, AdB, NvH all contributed and participated in the design of the study. IRP and NvH were mainly responsible for writing this manuscript, but all other authors critically reviewed the manuscript multiple times. All authors have given approval to the final manuscript
Name and contact information for the trial sponsor {5b}	The Department of Rheumatology at the Sint Maartenskliniek fulfils the role as sponsor and of coordinating study centre.
Role of sponsor {5c}	The GO TEST Finale is funded by ZonMw (grant number: 80-87600-98-19030). During the grant proposal process the study protocol has been peer reviewed by external reviewers. After awarding the grant, ZonMw had not influenced the study design and/or data collection.

## Introduction

### Background and rationale {6a}

Gout is a rheumatic disease characterized by the deposition of monosodium urate (MSU) crystals in synovial fluid and/or soft tissues during a prolonged state of hyperuricaemia [1]. Gout has different clinical presentations such as (recurrent) acute arthritis (gout flare), chronic arthritis, tophi, and structural joint damage. Pharmacological management of gout consists of flare treatment with anti-inflammatory medication, and long-term treatment with urate-lowering therapies (ULT) [1]. Serum urate levels are lowered below the solubility threshold for MSU to reverse MSU deposition so as to prevent new gout flares and/or to dissolve tophi.

Guidelines of major rheumatology societies recommend ULT using a lifelong treat-to-target (T2T) strategy [2–4]. In this treat-to-target (T2T) approach, serum urate is lowered by increasing the dose or combining ULT until the target of <0.36mmol/l (6mg/dl) or even <0.30mmol/l (5mg/dl) in patients with tophi, severe polyarticular gout and/or erosions is reached [3–6] and maintained at target. Although globally rheumatologists strongly believe in a continued T2T approach [7], an alternative strategy also commonly used in daily practice is a treat-to-avoid-symptoms (T2S) approach [8]. In this T2S strategy, ULT is solely dosed on symptoms, regardless of serum urate levels. This strategy is preferred by the American College of Physicians [9], since they state that the benefits of a T2T strategy do not outweigh the disadvantages (e.g. serum urate monitoring and medication escalation).

Although a T2T strategy has proven superior for gout patients in the intensification phase of ULT treatment [10], surprisingly, high-quality evidence regarding continued ULT use when remission has been achieved is lacking. It seems sensible to continue ULT for life, since gout is considered a chronic disease [1]. A previous systematic review on the discontinuation of ULT included five studies with gout patients [11]. These observational studies were performed between 1974 and 2011 and included 10–211 patients. Gout relapse rates after ULT cessation were 36-81% and gout flares occurred 1–4.5 years after ULT discontinuation.

In daily practice, ULT is often tapered or stopped in the absence of gout flares. A Dutch retrospective study showed that the non-persistence of allopurinol increased from 39 to 57% 1 and 5 years after ULT initiation [12]. Only 39% of patients reached medication possession ratios of ≥80% during the 5 years of follow-up. This latter is in line with a previous review, which showed an overall ULT adherence rate of 47% [13, 14]. Since there is no compelling empirical evidence to continue ULT indefinitely when remission has been achieved, the most recent gout management guideline of the American College for Rheumatology (ACR) states that tapering or discontinuation of ULT can therefore be discussed [3].

A few supportive arguments can be made for a T2S discontinuation attempt when remission has been achieved while using ULT. Firstly, gout flares occur in patients after a prolonged state of hyperuricaemia and subsequent accumulation of MSU crystals in the body. This MSU crystal load is reduced after a period of ULT treatment [15, 16], although it is not clear how long this period must be for protection from gout flare to occur. Secondly, a recent insight shows that hyperuricaemia and gout flares lead to epigenetic changes and adaptation of the innate immune system, which result in an increased pro-inflammatory state [17]. It was also shown that changes in the trained innate immune system influence the duration and intensity of gout flares [18]. These changes occur under the condition that the stimulus (hyperuricaemia and gout flares) is still present and will sustain for a certain period of time after disappearance of this stimulus. It can be hypothesized that this pro-inflammatory state is reset after prolonged time of remission (absence of gout flares and serum urate normalization due to ULT use) [19]. Gout patients who have been in remission for a longer period of time might therefore be in a state comparable to the period before the clinical signs of gout started. Although hyperuricaemia reoccurs in almost all patients after ULT discontinuation [11], this does not necessarily lead to recurrent gout flares, but may also result in asymptomatic hyperuricaemia.

In conclusion, a continued ULT T2T and T2S discontinuation strategy are both used in clinical practice in gout patients in remission. Although most rheumatologists strongly believe continuing a T2T strategy for life is expected to be superior to a T2S discontinuation strategy, in terms of gout control, the strategies have not been compared before in gout patients in remission. This unanswered question has been included in several (inter)national research agendas [20, 21]. We therefore designed a randomized trial with the objective to demonstrate the superiority of a T2T ULT continuation strategy compared to a T2S discontinuation ULT discontinuation strategy in gout patients in remission.

### Objectives {7}

#### Primary objective

The primary aim of the Gout TrEatment Strategy (GO TEST) Finale study is to investigate whether a continued ULT T2T strategy is superior to switching to a trial and error T2S ULT discontinuation strategy in gout patients currently using ULT and fulfilling preliminary gout remission criteria [22]. The between-group proportion of patients not in remission based on modified preliminary gout remission criteria during the last 6 months of 24-month follow-up is compared.

#### Secondary objectives

- To exploratory assess non-inferiority (only when superiority cannot be demonstrated) of the treat-to-symptom strategy compared to the treat-to-target strategy.
- To assess the between-group difference in the incidence (cumulative incidence and incidence density) of gout flares during the follow-up period of 24 months according to gout flare criteria [23].
- To assess the proportion of participants that require reintroduction of ULT in the T2S discontinuation strategy group.
- To assess the between-group difference in the use of ULT and gout flare medication (anti-inflammatory medication).
- To assess predictors for successful ULT cessation in the T2S discontinuation strategy group including clinical, radiological, immunological, and genetic variables.
- To evaluate the between-group difference in Patient-Reported Outcome Measures, by three monthly analysing the 5-level EuroQol-5 domains (EQ-5D-5L) questionnaire, functioning by using the Health Assessment Questionnaire Disability Index (HAQ-DI), numeric rating scale (NRS) for pain and NRS global health.
- To assess the between-group difference in types and frequency of adverse events, with a special focus on change in the renal function defined by Glomerular Filtration Rate Chronic Kidney Disease – Epidemiology Collaboration (GFR-CKD-EPI) and incidence of major adverse cardiovascular events (defined as arrhythmia, non-fatal stroke, non-fatal myocardial infarction, (hospitalization for) acute coronary syndrome, cardiovascular hospitalizations and cardiovascular death) during the follow-up period of 24 months. For the MACE there is a special interest in the first 3 to 6 months after ULT cessation and reintroduction of ULT in the T2S group.
- To assess the (between-group) difference in prescribed medication compared with refill rates during the follow-up period of 24 months.
- To assess the incremental cost-effectiveness of a T2T ULT continuation strategy compared to a T2S ULT discontinuation strategy.

### Trial design {8}

The GO TEST Finale study is an investigator-initiated pragmatic, multicentre, two-arm, randomized, open-label, superiority treatment strategy trial in gout patients with disease remission ≥ 12 months while using ULT (allopurinol, benzbromarone and/or febuxostat). Participants are randomized 1:1 to either the T2T ULT continuation strategy group or the T2S discontinuation strategy group and are followed for 24 months.

## Methods: participants, interventions and outcomes

### Study setting {9}

#### Study setting

The study started on 23 February 2021 and is currently recruiting patients in seven participating centres in the Netherlands (all rheumatology departments): Sint Maartenskliniek (Nijmegen, Geldrop, Boxmeer and Woerden), VieCuri Medical Centre, Bravis hospital, Bernhoven hospital, Erasmus Medical Centre, Maastricht University Medical Centre, Medisch Spectrum Twente, Ziekenhuisgroep Twente and Martini hospital. Additionally, three centres (Radboudumc, Rijnstate hospital and hospital Gelderse Vallei) refer eligible patients to the Sint Maartenskliniek Nijmegen. Patients who had been treated at any time by a rheumatologist, but have been referred back to their general practitioner because of achieved remission are also contacted to participate. The METC Oost-Nederland judged our study to be a low-intervention clinical trial with medicinal products according to the new European Clinical Trial Regulation (CTR).

### Eligibility criteria {10}

Participants ≥18 years with gout (clinical diagnosis and/or fulfilling the 2015 ACR-EULAR gout criteria [24], currently using ULT (allopurinol, benzbromarone and/or febuxostat) and achieved remission for at least 12 months based on preliminary gout remission criteria (Table 1) [22]), are eligible for inclusion.

**Table 1:** Criteria used to define gout remission, based on the preliminary gout remission criteria (all criteria must be met for the state of remission to be present) [22]

**Criteria**	**Baseline**
Gout flares	No gout flares in the past 12 months
Tophi	No visible tophi during a physical examination in the past 12 months
Serum urate target	All known values of the past 12 months are ≤0.36mmol/l
Pain due to gout	A score of <2 at baseline out 0–10. Where 0 is no pain at all and 10 worse pain possible
Patient global assessment of gout disease activity	A score of <2 at baseline out 0–10. Where 0 is no activity at all and 10 worse disease activity possible

Regarding the preliminary gout remission criteria, originally de Lautour et al [22], stated that measurements of pain and patient global assessment should be measured at least twice over the last 12 months and measurements should be at equal distances apart. Since these assessments are not registered standardly at an inpatient clinic visit in our target population, we only used the baseline measurement as a criterion. In addition, the Likert scale/NRS scale for pain and disease activity is originally described as a 1–10 scale [22]. As we also use the validated gout flare criteria by Gaffo et al [23] during the study which includes an 11-point scale to score pain in rest (0–10) we also use the 0–10 scale for our remission criteria, to not confuse the participants with two different scales.

#### Exclusion criteria

Patients are excluded if any of the following criteria is met:

- Tophaceous gout (visible on physical examination at baseline)
- ULT use for any other indication than gout (for example nephrolithiasis)
- Strong contra-indication for all three of the most used gout flare treatment options (glucocorticoids, non-steroidal anti-inflammatory drugs (NSAIDs) and colchicine), as this hampers gout flare treatment
- Chronic use of glucocorticoids, and/or colchicine and/or interleukine-1 inhibitors for any diagnosis indication and/or regular use of NSAID for gout prevention or treatment.
- Anticipated follow-up time too short, e.g. life expectancy <2 years, or planned relocation out of reach of the study centre
- Not being mentally competent or insufficient knowledge of the Dutch language
- History of myocardial infarction or stroke in the past 6 months and/or congestive heart failure and/or a New York Heart Association (NYHA) class ≥III

This last exclusion criterion was added at the request of the METC Oost-Nederland after analysis of the CARES study [25] and since this group of patients had been excluded from the FAST study [26].

### Who will take informed consent? {26a}

Potential eligible participants are selected based on the above-mentioned inclusion and exclusion criteria using information from the electronic health record. They will be approached on behalf of their treating rheumatologist or general practitioner, using a letter accompanied by the patient information sheet and the informed consent form. After one to 2 weeks patients are contacted by the research physician or nurse by telephone to discuss the study and answer any questions. During the inclusion visit, written informed consent is obtained by the research physician or research nurse.

### Additional consent provisions for collection and use of participant data and biological specimens {26b}

A sub-study is optional for patients in the T2S discontinuation group of the study at the location Sint Maartenskliniek Nijmegen. Additional consent is requested for obtaining blood samples three-monthly consisting of a serum tube (10 ml), plasma (one citrate 10 ml and two ethylenediaminetetraacetic acid (EDTA) 2.7 ml) and a PAXgene (2.5 ml) tube to collect ribonucleic acid (RNA). All participants are also requested to give permission to approach them for future research.

## Interventions

### Explanation for the choice of comparators {6b}

Since we hypothesize that a T2T ULT continuation strategy is superior compared to a T2S ULT discontinuation approach in gout patients in remission, the control group is the T2S discontinuation strategy group.

In the T2S strategy, ULT is tapered to stop and restarted in case of recurrent or persistent flaring or the emergence of tophi. Thereafter, (re)treatment is T2T, thus also based on serum urate levels. ULT is tapered to stop in a maximum of 6 weeks based on ULT dosage at baseline (Table 2). A taper strategy was recommended over a direct stop strategy by the regional medical review board. This was based on studies that showed associations between cardiovascular events and the start/stop of ULT, presumably due to rapid serum urate changes. Tapering is expected to prevent rapid serum urate changes and thereby serious cardiovascular adverse events. Two weeks after ULT cessation an extra blood sample is obtained to check for any changes in blood counts and kidney and liver function. Serum urate levels will most likely increase, but this is not acted upon as in the T2S discontinuation strategy, ULT treatment is based only on symptoms. A telephone consult is planned three weeks after ULT cessation to verify that ULT has indeed been stopped, to discuss the tapering process and to discuss the blood results.

**Table 2:** Tapering schedules of urate-lowering therapies in the treat-to-avoid-symptoms discontinuation group

**Baseline dose**	**First tapering step at baseline**	**Second tapering step 2 weeks from baseline**	**Third tapering step 4 weeks from baseline**	**Fourth tapering step 6 weeks from baseline**
**Allopurinol**				
50–100 mg/day	Stop	-	-	-
150–300 mg/day	100 mg/day	Stop	-	-
350–600 mg/day	300 mg/day	100 mg/day	Stop	-
≥ 650 mg/day	600 mg/day	300 mg/day	100 mg/day	Stop
**Benzbromarone**				
50 mg/day	Stop	-	-	-
100 mg/day	50 mg/day	Stop	-	-
200 mg/day	100 mg/day	50 mg/day	Stop	-
300 mg/day	200 mg/day	100 mg/day	50 mg/day	Stop
**Febuxostat**				
40 mg/day	Stop	-	-	
80 mg/day	40 mg/day	Stop	-	
120 mg/day	80mg/day	40mg/day	Stop	

**Fig. 1:**
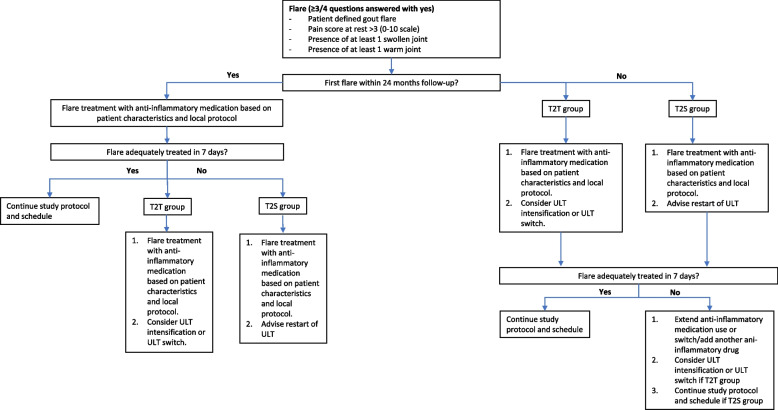
Gout flare protocol. ULT, urate-lowering therapy; anti-inflammatory medications: colchicine, NSAIDs, and/or glucocorticoids

A gout flare during ULT tapering or after discontinuation is treated with anti-inflammatory drugs (colchicine, NSAIDs and/or glucocorticoids) based on patients’ previous experiences, comorbidities, co-medications and local (centre-specific) flare treatment protocol (Fig. 1). In case of a first gout flare, ULT is not restarted yet, but in case a second gout flare occurs at any time during follow-up, or in case a flare persists for more than 7 days despite anti-inflammatory treatment, ULT restart is advised.

In case of a restart, the ULT dosage is stepwise increased in approximately 4–8 weeks until the baseline dosage has been reached. Gout flare prophylaxis (based on the patient’s previous experiences, comorbidities and co-medications) for at least three months is advised while ULT is restarted. From this point forward a continued T2T strategy is followed again since it is desirable to achieve remission quickly to prevent new complaints and/or possible joint damage. We chose to not immediately restart the ULT at the first gout flare in consultation with our patient partners when designing this trial. They stated that the benefit of not using ULT outweighs the disadvantages of a gout flare and therefore one (short) gout flare is accepted.

### Intervention description {11a}

In the intervention group, ULT treatment will be continued using a T2T strategy in which ULT is dosed based on serum urate levels as well as symptoms. Since visible tophaceous gout at baseline is an exclusion criterion for participation, we chose a uniform treatment target of <0.36mmol/l for the T2T ULT continuation group, with a lower limit of 0.20 mmol/l (0.34 mg/dl). Patients who have had tophi previously are treated using the treatment target of serum urate <0.36 mmol/l. The lower limit was chosen due to suggestions of a correlation between low uric acid and neurodegenerative diseases [27, 28]. Therefore, in case serum urate level drops below 0.20 mmol/l during follow-up, a dose reduction is considered and, in case serum urate level becomes higher than 0.36 mmol/l and/or gout flares occur ULT treatment is intensified. This latter can be done by increasing dosage, combing or switching ULT until the serum urate target and/or clinical remission are achieved again. When side effects occur, ULT will be switched as well

All treatment decisions are ultimately at the discretion of the patient and the treating physician.

### Criteria for discontinuing or modifying allocated interventions {11b}

Tapering or stopping of ULT in the T2T ULT continuation group (intervention group) will only be done when serum urate level drops below 0.20mmol/l, side effects, or toxicity occurs. As patients included in the study have been stable on ULT for at least 12 months, it is expected that few changes in ULT will have to be made during the study period.

In the T2S ULT discontinuation group (control group), ULT is restarted in case of recurrent flaring or at the emergence of tophi. However, if a first gout flare is severe, an immediate restart of ULT is an option. Finally, if any other unforeseen circumstances occur and this can be linked to the ULT discontinuation period, the re-introduction of ULT can be considered.

### Strategies to improve adherence to interventions {11c}

Therapy and protocol adherence of all participants is monitored during follow-up for both groups. Medication use (ULT and anti-inflammatory medication) is monitored through three-monthly questionnaires. Patients in the T2S discontinuation group are contacted after planned ULT discontinuation to verify if medication is indeed stopped.

If patients experience a gout flare they are encouraged to contact the outpatient clinic. The research physician/nurse or student assistant verifies if the self-reported gout flare is in accordance with the gout flare criteria; if there is any discrepancy in those results or doubt exists if the current event is indeed a gout flare, an extra outpatient visit is scheduled to assess the signs and symptoms. If the questionnaire registers a gout flare which has not been reported by telephone, the research physician /research nurse will contact the patient and will consider therapy adjustments.

### Relevant concomitant care permitted or prohibited during the trial {11d}

There are no prohibitions for the use of other medicines. Patients with chronic use of colchicine, NSAIDs (for gout activity), glucocorticoids and/or interleukin-1 inhibitors for gout or any other medical condition are not eligible for inclusion, but when this treatment is started during the study it can be continued. Use of co-medication will be registered throughout the study.

### Provisions for post-trial care {30}

Our intent is to perform a 3-year observational extension of the original 2-year study period. Patients are allowed to switch treatment strategy after the trial ends. Treatment decisions after the study ends will be made by the patient and treating physician, and are unrestricted.

### Outcomes {12}

#### Primary outcome

The primary outcome is the between-group difference in the proportion of patients not fulfilling a modified version of the preliminary remission criteria for gout [22] (no tophi, no gout flares, NRS pain due to gout <2, NRS gout disease activity <2) over the last 6 months of 24-month follow-up. The modification consists of omitting the serum urate target <0.36 mmol/l prerequisite, as the value of this is a surrogate outcome measure and is indeed the very question to study whether this is related to clinical outcomes (Table 3).

**Table 3:** criteria used to define gout remission, based on modified preliminary gout remission criteria (all criteria must be met for the state of remission to be present) [22]

**Criteria**	**Primary outcome at month 24**
Gout flares	No gout flares in the past 6 months of the total follow-up period
Tophi	No visible tophi during the total follow-up period
Pain due to gout	A NRS score of <2 during the past 6 months of the total follow-up period on a 0-10 scale. Where 0 is no pain at all and 10 worse pain possible
Patient global assessment of gout disease activity	A NRS score of <2 during the past 6 months of the total follow-up period on a 0–10 scale. Where 0 is no activity at all and 10 worse disease activity possible

#### Secondary outcomes

See secondary objectives for an overview of objectives and their derived outcomes.

Our hypothesis is that the T2T continuation ULT strategy is superior at the group level in ULT using gout patients in remission. However, this advice might be personalized. Potential predictors, including clinical, immunological, (epi)genetic and imaging variables collected during the GO TEST Finale study, are studied as potential effect modifiers. This will be done by exploratory prediction modelling using logistic and cox regression modelling including all candidate predictors, thereafter, followed by the development of a prediction model and subsequent internal validation using k-fold cross-validation.

To investigate the cost-effectiveness of both treatment strategies, the costs of either approach is calculated and will be compared with each other. Current medication use (type, dosage, frequency and administration route) will be recorded and updated at each clinic visit. Costs of medication use will be derived from the Dutch formulary and increased with a pharmacist charge. Self-reported healthcare resource usage and productivity loss will be assessed using questionnaires administered at baseline and then three monthly until the study end at 24 months. Medical consumption will be assessed using the iMTA Medical Consumption Questionnaire (iMCQ) and productivity loss will be assessed by using the iMTA Productivity Cost Questionnaire (iPCQ). Cost prices will be calculated according to the 2016 Dutch guideline for health economic evaluation [29]. A friction cost will be applied to estimate the productivity losses as defined in the Dutch costing manual, and based on the reference costs of being unable to perform paid or unpaid work.

Incremental cost-effectiveness ratios (ICER) will be calculated with credible intervals using bootstrapping. Incremental net monetary benefit (iNMB) will be estimated based on different levels of willingness-to-pay (WTP).

### Participant timeline {13}

**Fig. 2:**
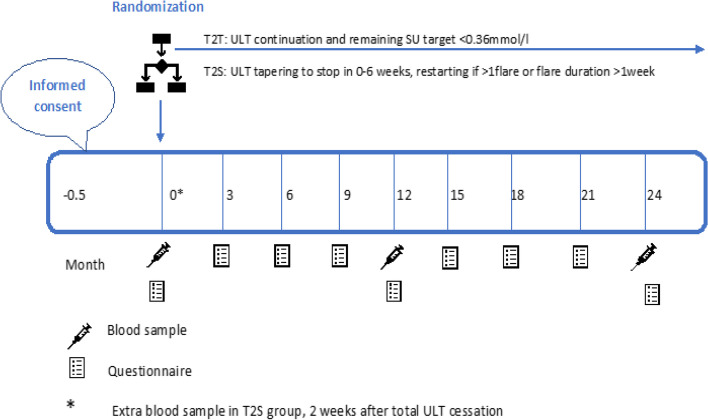
Participation timeline

During follow-up, three hospital visits are scheduled at the outpatient clinic, at baseline, month 12 and month 24 for both treatment strategy groups. For patients randomized to the T2S discontinuation group, 2 weeks after complete ULT cessation an extra blood sample is obtained and a telephone consult is scheduled one week later to discuss results and make sure patients indeed stopped the ULT (Fig. 2). Questionnaires are sent monthly (gout flare questionnaire) and three monthly (other questionnaires).

### Sample size {14}

The null hypothesis is that no statistical difference is found between the continued T2T approach and the T2S discontinuation approach. Since the T2S group receives less intensive treatment, the superiority of the T2S discontinuation approach in terms of efficacy is considered unlikely and also irrelevant. After all, one would prefer the less intensive treatment as long as the more intensive treatment is not clearly superior, regardless of whether the less intensive treatment is equally effective or superior. Therefore, we use a one-sided test to achieve the same power to show the superiority of continued T2T with a smaller study size compared to a two-sided test with the same alpha.

Based on earlier studies and on unpublished data from our own cohort (2400 gout patients from Sint Maartenskliniek, Radboudumc and Rijnstate) we estimate that 8% of gout patients following the treat-to-target strategy experience at least 1 gout flare during the 2-year follow-up and 4% of patients are not in remission in the last 6 months of the 24-month follow-up. For the patients in the treat-to-symptom group, less data is available, but we estimate that 12% are not in remission in the last 6 months of the follow-up time, based on the 5 smaller cohort studies included in the review of Beslon et al [11].

Using alpha 0.05, power 1-beta 0.80, one-sided superiority testing with a two proportion *z* test, and expected proportion of patients not fulfilling the adapted preliminary remission criteria for gout domains during the last 6 months of the study of 0.04 and 0.12 in the T2T strategy and control group T2S strategy (difference 0.08, number needed to harm, number needed to harm, NNH, 12), 278 analysable patients are needed. Accounting for 10% dropout, 310 patients will be included. If during follow-up dropout turns out to be lower (based on incidence density of dropout/patient-years), fewer patients will be included.

Only when superiority is not shown, non-inferiority (NI) of T2S compared to T2T will also be tested (using a NI margin of 8%, based on the NNH of a maximum of 12). Assuming 278 evaluable patients, point estimates for the primary outcome of 0.04 and 0.05 in the intervention and control group, and one-sided alpha of 5%, the power for proclaiming non-inferiority will be around 87%. However, we are aware of a higher risk of a false-positive result, also because this secondary analysis uses an opposite hypothesis of the original superiority hypothesis, therefore the non-inferiority analysis is considered an exploratory secondary analysis.

The NI margin is just like the difference in the superiority analysis based on the notion — developed together with patient partners — that a lower limit of NNH of 12 (not obtaining or loss of gout remission) balances nicely with the numbers needed to benefit of between 2 and 4 of not needing daily ULT pills for up to 2 years. In other words, a treatment effect smaller than an 8% point difference is not considered worthwhile compared to the burden of taking the medication.

### Recruitment {15}

Participants are recruited at the Rheumatology department of various hospitals. Currently, we aim at the participation of seven Dutch centres in this trial.

## Assignment of interventions: allocation

### Sequence generation {16a}

Participants will be randomized in a 1:1 ratio to either the continuation T2T or T2S discontinuation strategy. Participants will be randomized by the research physician using a computerized randomization procedure with variable block sizes. No stratifications are made since no strong predictors of flare were found in the previous literature

### Concealment mechanism {16b}

Opaque-sealed envelopes are used to conceal the allocation sequence prior to the randomization of each patient.

### Implementation {16c}

Randomization blocks are generated by an online programme and have variable block sizes to achieve the intended allocation ratio and to prevent the allocation from being predictable for the treating physician..

## Assignment of interventions: blinding

### Who will be blinded {17a}

Patients, research nurses and physicians will not be blinded for treatment allocation. Analyses will be performed blinded for treatment groups. Due to the nocebo effect, patients (and treating physicians) in the discontinuation treat-to-avoid-symptom group could expect more gout flares after ULT cessation, and therefore the number of gout flares could be higher in this group. However, validated gout flare criteria are used to determine whether or not an event is deemed as a gout flare or not. If in doubt an out-patient visit is scheduled for a clinical examination by a (research) physician

### Procedure for unblinding if needed {17b}

Does not apply.

## Data collection and management

### Plans for assessment and collection of outcomes {18a}

**Fig. 3:**
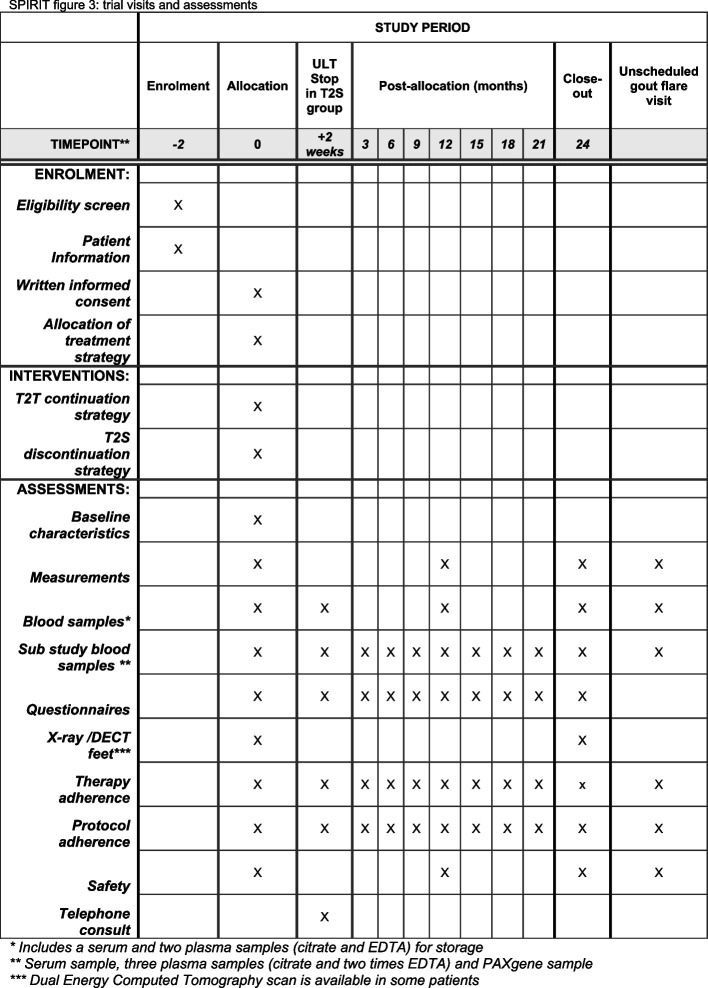
SPIRIT: trial visits and assessments

Regular visits are planned at baseline, month 12 and month 24 (Fig. 3). Digital or paper questionnaires are sent monthly (gout flare questionnaire) and three monthly (set of questionnaires). The Health Assessment Questionnaire (HAQ)-II, iMTA Productivity Cost Questionnaire (iPCQ), iMTA Medical Consumption Questionnaire (iMCQ), EuroQol-five-Dimensional Questionnaire with Likert response(EQ-5D-5L) are all validate questionnaires. Based on the advice of the OMERACT for study outcomes for acute gout we included the HAQ-II. The use of the EQ-5D-5L, iPCQ and iMCQ for this study was requested and granted.

### Plans to promote participant retention and complete follow-up {18b}

Firstly, a telephone call is scheduled 2 weeks after ULT cessation in the T2S discontinuation group to make sure ULT is indeed stopped. Secondly, a process database is maintained to make sure questionnaires and outpatient visits are scheduled on time and have been completed. If a questionnaire has not been returned on time (digital or by post), the participant is requested by email or telephone call, to complete it as soon as possible. Also, participants have a direct work phone number of the physician-researcher so that contact can be made if they are experiencing a gout flare or have any questions. If participants are lost to follow-up or withdraw consent, the data that has been collected up to that time will be used

### Data management {19}

Patient, disease, and treatment characteristics are extracted from the electronic health record and encrypted entered and stored in the data management system Castor EDC. Questionnaires are sent and answers are locked in the database using automated sending and data saving through Castor EDC. If patients receive their questionnaires by post, the answers are entered manually and locked afterwards. All paper questionnaires are stored at the concerned study site. A data management plan is created in line with the General Data Protection Regulation, which can be viewed upon request.

### Confidentiality {27}

The collected data will be coded and stored by the rules of good clinical practice. Personal data will be handled in compliance with the Dutch Act on Implementation of the General Data Protection Regulation (Algemene Verordering Gegevensbescherming, AVG). Data of participants will be handled confidentially. A unique study code will be assigned to each participant. The code consists of an abbreviation for the study centre and a number. Patients’ name and contact details will be saved together with the unique patient code in the process database (ID-log) which will be secured with a password. The password is available to the coordinating investigator, principal investigators, and study team members when this is necessary to execute their tasks and study monitor.

### Plans for collection, laboratory evaluation and storage of biological specimens for genetic or molecular analysis in this trial/future use {33}

The blood samples that are collected for regular care throughout the study will not be stored and will be handled according to local laboratory agreements that conform to regular care. Blood samples obtained for the biobank will be stored for future research and can be stored for a maximum of 15 years. Written informed consent is obtained to do so.

## Statistical methods

### Statistical methods for primary and secondary outcomes {20a}

#### Primary outcome

For superiority testing data is handled primarily by intention-to-treat analyses. Analyses will be conducted using the two proportion *z* test. Results will be interpreted with a *p*-value of *P* <0.05 as significant.

#### Secondary outcomes

Only when superiority cannot be demonstrated, non-inferiority testing will take place exploratory of the treat-to-symptom strategy compared to the treat-to-target strategy by calculating a confidence interval using the Miettinen and Nurminen method [30].

- The between-group difference in the incidence (cumulative incidence and incidence density rate) of gout flares during the follow-up period of 24 months.

Cumulative flare incidence is expressed as a proportion of the number of patients experiencing a flare at any time during the follow-up period. This is calculated separately for both groups and will be described by means of descriptive statistics as *n* (%). These proportions are compared using a chi-squared test or Fisher’s exact test

- The proportion of participants that require reintroduction of ULT in the T2S group during the 24-month follow-up period.

The proportion of the number of patients in the T2S group in which the ULT is restarted will be expressed as n (%).

- The between-group difference in SU change during the total follow-up time and particularly at baseline and at the end of follow-up at 24 months.

Serum urate at baseline serum urate, 2 weeks after ULT discontinuation, month 12 and month 24 will be described as mean and 95% confidence intervals (CI). The difference from baseline measurement will also be described by the mean and 95% CI. A difference between the two groups will be compared with an independent *t*-test or Mann-Whitney test.

- To evaluate the between-group difference in Patient-Reported Outcome Measures (PROMs), by three monthly analysing the EQ-5D-5L questionnaire, functioning by using the Health Assessment Questionnaire Disability Index (HAQ-DI), numeric rating scale (NRS) for pain and NRS global health

Descriptive statistics in the form of mean and 95% CI will be used. The difference from baseline will also be presented using the mean and 95% CI. The difference in change in EQ-5D-5L, HAQ-DI, NRS pain and NRS global health between the groups will be analysed by an independent t-test or Mann-Whitney test.

- To assess the between-group difference in types and frequency of adverse events, with a special focus on change in renal function (CKD-EPI) and incidence of cardiovascular events during the follow-up period of 24 months.

During the study, adverse events are registered using the Common Toxicity Criteria for Adverse Events. The results will be presented by using descriptive statistics. The occurrence of adverse events between the groups is compared using a chi-squared test or Fisher’s exact test.

- To assess the between-group difference in use of ULT and flare medication (colchicine, NSAIDS and/or glucocorticoids).

Per group, the proportion of patients using flare medication, restarting ULT or experiencing a ULT increase/decrease will be described as *n* (%). The difference between the groups will be analysed using a chi-squared test or Fisher’s exact test.

- To assess the between-group difference in prescribed medication compared with refill rates and self-reported adherence during the follow-up period of 24 months.

First, it will be examined per group whether the self-reported medication use corresponds with the refill adherence information from the pharmacy/LSP, this is described by means of *n* (%). Then the difference between the two treatment groups will be compared by using a chi-squared test or Fisher’s exact test.

- To assess predictors for successful ULT cessation in de T2S strategy group including clinical, radiological, and immunological, variables.

Regression analyses will be used to exploratory identify possible predictive variables for unsuccessful withdrawal of uric acid-lowering medication. This will include radiological, biochemical, patient and disease characteristics.

### Interim analyses {21b}

Not applicable, no interim analyses for safety, efficacy or futility will be performed. Safety within the trial will be monitored by an installed Data Safety Monitoring Board (DSMB).

### Methods for additional analyses (e.g. subgroup analyses) {20b}

Not applicable.

### Methods in analysis to handle protocol non-adherence and any statistical methods to handle missing data {20c}

We follow the guideline in handling missing data [31]. When it concerns missing completely at random (MCAR) or missing at random (MAR), multiple imputation will be used. Imputing then will always increase the precision and often also reduces bias. However, when data are missing not at random (MNAR), best-worse and worse-best sensitivity analyses will be added. For missings in the continued T2T group, this would mean replacing missing values for continued remission in the best-case scenario and no remission values in the worst-case scenario.

### Plans to give access to the full protocol, participant-level data and statistical code {31c}

The full protocol is available at https://zorgevaluatienederland.nl/evaluations/go-test-finale. The data collected during the study will be available at reasonable request.

## Oversight and monitoring

### Composition of the coordinating centre and trial steering committee {5d}

The Department of Rheumatology at the Sint Maartenskliniek fulfils the role as sponsor and of coordinating study centre. The day-to-day work will be done by a research physician (IRP). The trial steering committee consist of three additional rheumatologists (NvH, MF and AdB).

### Composition of the data monitoring committee, its role and reporting structure {21a}

The Data Safety Monitoring Board (DSMB) consist of the researchers of the Sint Maartenskliniek (IRP, NvH and AdB) to inform the other members about the progression. The two independent members are dr. M. Martens (rheumatologist at the Sint Maartenskliniek, he has no additional role in the trial) and dr. LM. Verhoef (researcher at the Sint Maartenskliniek, she has no additional role in the trial). The DSMB will convene 2–3 times a year. The advice(s) of the DSMB will be sent to the sponsor of the study. Should the sponsor decide not to fully implement the advice of the DSMB, the sponsor will send the advice to the reviewing METC Oost-Nederland, including a note to substantiate why (part of) the advice of the DSMB will not be followed

### Adverse event reporting and harms {22}

Adverse events (AE) are defined as any undesirable experience occurring to a subject during the study, whether or not considered related to the trial procedure. All AEs reported spontaneously by the subject or observed by the research physician, or his staff will be recorded. A serious adverse event (SAE) is any untoward medical occurrence or effect that; results in death; is life-threatening (at the time of the event); requires hospitalization or prolongation of existing inpatients’ hospitalization; results in persistent or significant disability or incapacity or any other important medical event that did not result in any of the outcomes listed above due to medical or surgical intervention but could have been based upon appropriate judgement by the investigator. Elective hospital admissions will not be considered as a SAE.

The research physician of the Sint Maartenskliniek will report the SAEs through the web portal to the accredited METC Oost-Nederland within 7 days of first knowledge for SAEs that result in death or are life-threatening followed by a period of a maximum of 8 days to complete the initial preliminary report. All other SAEs will be reported within a period of a maximum of 15 days after the sponsor has first knowledge of the serious adverse events. All AEs will be followed until they have abated, or until a stable situation has been reached. Depending on the event, follow-up may require additional tests or medical procedures as indicated, and/or referral to the general physician or a medical specialist.

In addition, a safety report is sent to the accredited METC Oost-Nederland once a year.

### Frequency and plans for auditing trial conduct {23}

According to the ‘richtlijn kwaliteitsborging mensgebonden onderzoek’ from the NFU (Dutch Federation of University Medical Centers (NFU), dating March 2019, this study can be classified as a study with negligible/low risk. However, to ensure monitoring of planning, progress and outcomes of the study, a Data Safety Monitoring Board (DSMB) will be installed which reviews data on recruitment, safety, protocol adherence, protocol updates and results of monitoring visits. In the Castor EDC database, an automatic digital audit trail is kept and can be consulted if needed.

### Plans for communicating important protocol amendments to relevant parties (e.g. trial participants, ethical committees) {25}

Important protocol amendments, such as adding a participating centre, will be reported and approval will be requested of the METC Oost-Nederland. If any important protocol amendments are made which alter the current eligibility criteria, study procedures or outcomes, all participating centres will be informed by letter and email.

### Dissemination plans {31a}

Results will be available through scientific publications and oral presentations. At the end of the study, a summary will be submitted at the central and regional METC Oost-Nederland. All participants will receive a dutch lay summary of the results.

## Discussion

With this multicentre, randomized, superiority trial, we aim to investigate whether a continued T2T strategy with ULT is superior compared to a trial and error T2S discontinuation strategy for gout patients, who are in remission while using ULT. We would like to highlight and discuss some aspects of the design in more detail below.

First, our hypothesis is superiority on the group level of a ULT T2T continuation strategy compared to switching to a ULT discontinuation T2S strategy in gout patients in remission. The review by Beslon et al [11] showed a wide range of gout flare relapse rates in different cohort studies. Possible predictive factors (all not statistically significant) for unsuccessful ULT discontinuation (defined as recurrence of gout) were: higher serum urate levels before and after ULT treatment, higher body mass index (BMI), onset of gout at a young age and longer duration of ULT treatment. Another prospective cohort study found low serum urate levels while using ULT (<0.30 mmol/l or <5.05 mg/dl) and after ULT cessation (<0.51 mmol/l or <8.75 mg/dl), were significantly associated with the longest period of gout-free remission [32]. This led to the suggestion that an intermittent ULT strategy might work for some gout patients. Potential predictors including clinical, immunological (epi)genetic and imaging variables will be studied as effect modifiers to explore if personalized treatment advice can be given.

Effects on CVD and renal function are also important, even though they are not the initial reason to use ULT. Gout is strongly associated with cardiovascular disease (CVD): patients with gout often have traditional risk factors for CVD, and patients with CVD often have gout. Also, tophi can sometimes form in coronary and carotid arteries [33, 34]. Both hyperuricaemia and gout are also independent risk factors for all-cause mortality and cardiovascular mortality. The exact mechanism for this is not yet known. Because high serum urate is associated with CVD, it seems logical that lowering serum urate would result in a reduced risk of CVD. Data however is conflicting: the FREED trial did show a reduction in CVD and in fact all-cause mortality when comparing febuxostat with placebo [35]. On the other hand, in the large ALL-HEART study [36], no protective effect of allopurinol compared to usual care was found in ischemic heart patients (without gout) with regard of non-fatal myocardial infarction, non-fatal stroke, or cardiovascular death. Complicating factor is that some data suggest that both starting and stopping ULT can indeed increase CVD risk. A subanalysis of the CARES study [37] showed a mortality increase after the cessation of ULT and an observational cohort study showed increased cardiovascular morbidity after initiation and discontinuation of ULT [38]. Based on the current evidence (and at the advice of our METC Oost-Nederland) taper schedules instead of direct withdrawal were implemented in the T2S ULT discontinuation group. Also, patients with severe heart failure and/or recent cardiovascular events were excluded from participation, since this group was also not included in the FAST and CARES study. It remains to be seen what the effect of ULT trial and error stopping means for cardiovascular risk and, as this strategy is widely used, any signal either detrimental or beneficial would be of great importance

We chose a pragmatic approach with regard to tophaceous gout. Only if tophi are visible on physical examination at baseline, participants are excluded from participation, since the urate load has not been dissolved properly yet and it is expected that some local inflammation is present contributing to the risk of joint damage in this group. If tophi have been present in the past, but were absent for at least 12 months we deemed the urate load to be sufficiently lowered to be able to participate in the study.

Another point to address is that our study is not blinded and/or placebo controlled. Blinding of participants and physicians would perhaps be ideal and would perhaps provide a better picture of the biological efficacy of the interventions under ideal conditions, and would reduce the chance of a false positive trial due to expectation bias as T2S patients who stop ULT might anticipate flares more strongly than continuing patients. Also, physicians might be more likely to restart ULT in case of a flare visit in the presence of elevated serum urate levels. However, we designed a pragmatic trial and focused on the question of which treatment strategy is most effective in daily clinical practice. Since, in daily practice, patients and physicians do know which strategy they follow, blinding patients could give a distorted picture of the (cost) effectiveness in daily clinical practice. Furthermore, arthritis is also objectively scored by the physician and is part of both the gout flare and preliminary gout remission criteria. Acute inflammatory gout is in general fairly distinguishable from non-inflammatory symptoms. Lasty, a blinded and placebo-controlled trial would be very complicated and unfeasible, as blinding of treatment would also require blinding of the serum urate levels, making tapering and restarting complex, perhaps almost impossible since you would want to see the lab results before prescribing anti-inflammatory medication. Also, three different types of ULT are included, with different dosages, which further complicates the use of a placebo. And finally, it would also result in a prohibitive cost increase.

A final topic to discuss is the 2-year study duration. The choice for 2 years was based on previous research showing that most gout flares occur 1–4.5 years after ULT cessation. Therefore, a 2-year study would give a reasonable picture of whether T2T indeed has merit over T2S at least for this timeframe. However, an extension of up to 5 years is certainly desirable and is currently being designed since this would give a completer picture of flare rate over time in the T2S group and this would also provide more follow-time for adverse events.

In conclusion, continued T2T and T2S discontinuation ULT strategies are both used in clinical practice, with their own possible pros and cons. Although continuing the T2T strategy lifelong is mostly thought to be superior to a T2S discontinuation strategy, this has not been determined before in gout patients in remission. This randomized superiority trial will add important and unique knowledge to deliver optimal gout care and will also gain insight into the association of ULT discontinuation and cardiovascular and renal events.

## Trial status

Recruitment started on 23 February 2021 and recruitment is still ongoing according to protocol version 11.0 23-01-2023. It is estimated that recruitment will be finished in spring 2023, and the last patient out visit is expected in spring 2025.

## Data Availability

Data will be handled according to FAIR principles. After completion of the study, data will be available on reasonable request by contacting the corresponding author. The request will be reviewed by the trial steering committee.

## Abbreviations

ACR: American College for Rheumatology
AE: Adverse event
AVG: Algemene Verordering Gegevensbescherming / General Data Protection Regulation
BMI: Body mass index
BROK: Basiscursus Regelgeving en Organisatie voor Klinische Onderzoekers
CTR: Clinical Trial Regulation
CVD: Cardiovascular disease
DECT: Dual-energy computed tomography
DSMB: Data Safety Monitoring Board
E-CRF: Electronic Case Report Form
EDTA: Ethylenediaminetetraacetic acid
EULAR: European Alliance of Associations for Rheumatology
EQ-5D-5L: EuroQol-five-Dimensional Questionnaire
GFR-CKD-EPI: Glomerular Filtration Rate Chronic Kidney Disease – Epidemiology Collaboration
HAQ-DI: Health Assessment Questionnaire Disability Index
ICER: Incremental cost-effectiveness ratio
ICTRP: International Clinical Trial Registry Platform
INMB: Incremental net monetary benefit
MAR: Missing at random
MCAR: Missings completely at random
MCQ: Medical Consumption Questionnaire
METC: Medical Ethical Review Board
MNAR: Missings not at random
MSU: Monosodium urate
NFU: Dutch Federation of University Medical Centers
NI: Non-inferiority
NNH: Number needed to harm
NRS: Numeric rating scale
NSAID: Non-steroidal anti-inflammatory drugs
NYHA: New York Heart Association
PCQ: Productivity Cost Questionnaire
RNA: Ribonucleic acid
SAE: Serious adverse event
ULT: Urate-lowering therapy
WTP: Willingness to pay
T2S: Treat-to-avoid-symptoms
T2T: Treat-to-target
